# Progress in Medicine

**Published:** 1896-04-25

**Authors:** 


### Progress in Medicine.

### DIABETES.

**Pathology.**—Professor Carl Von Noorden's[1] lecture on "Certain Forms of Glycosuria, with special reference to Alimentary Glycosuria" is particularly valuable, not so much for any new facts that it contains, but because he succinctly indicates how far we are warranted in accepting the many hypotheses that have been advanced in regard to glycosuria. At the outset he remarks that when we find that a person, after cerebral apoplexy, passes grape sugar with the urine for several hours a day, when we find that in bad cases of poisoning with morphine, chloralamide, carbonic oxide, strychnine, and other substances, the urine contains sugar, we are *not* able at present to state with authority that the disturbances at work in the body are utterly distinct from the disorders in diabetes. That the processes are different is but a supposition founded on the fact that such acute irregularities in the way the sugar is disposed of hardly ever develop into that well-known chronic disease, diabetes mellitus. The likelihood of their being distinct is further increased when we note that transitory glycosuria in animals is effected by similar causes as in human beings (injury to nerves and poison), while the chronic diabetes in man is reproduced by quite another experiment, bearing no relation to nerve irritation (extirpation of the pancreas).

Noorden groups the types of glycosuria as follows: 1. Cases due to hyperglycæmia, the consumption of sugar being normal, viz., (*a*) alimentary glycosuria; (*b*) glycosuria from excessive production of sugar from glycogen (traumatic and toxic forms). 2. Cases due to alteration in the filtering action of the kidneys, *e.g.*, in phloridzin poisoning, this substance causing the kidneys to greedily extract the sugar from the blood and making it pass into the urine. The blood thereby grows poor in sugar, and in order to replace this important constituent, new sugar is formed with great energy out of glycogen and albumin. Thus the over-production of sugar is here a result of the glycosuria. 3. Cases due to diminished consumption of sugar, whereby the blood comes to contain more than 0·2 per cent. of sugar, when the kidneys cease to act as a barrier to the sugar.

**Proportion of Sugar in Normal Urine.** — Noorden states that minute traces of sugar are found in the urine of every healthy person, but that it is doubtful whether these traces are passed through the epithelia of the glomeruli with the aqueous portion of the urine, or whether they originate later on from destroyed epithelial nuclei or from mucin. We find in every urine the chemical descendants of cells, especially of their nuclei, in the shape of nucleo-albumins; and A. Kössel has recently proved that carbo-hydrate can be formed by the decomposition of nucleo-albumin, or of nuclein, while we have long known that mucin contains a molecule of hydrate of carbon that is easily detachable. Then it must be admitted as possible that the traces of sugar in normal urine have originated later from decomposition of mucin and nuclein; while Baisch, in 1894, showed that normal urine contains a substance resembling dextrin, and at least one other kind of carbo-hydrate.

The starch molecule ("polysaccharid") is split up by the diastase of saliva and pancreatic juice in several carbohydrate molecules of smaller size ("monosaccharids" and "disaccharids"), and there results amidulin, erythrodextrin, achroödextrin, isomaltose, maltose; these, on entering the walls of the intestine and portal vein are almost entirely converted into grape sugar, dextrin and maltose meeting with the same fate if they happen to be contained in the food ingested. Cane-sugar is a disaccharid, and in the intestinal canal is split up by acids, ferments, and bacteria, into its two components, fruit sugar (levulose) and grape sugar (dextrose), and then passes into the blood; only when consumed in great amounts does cane sugar enter the blood as saccharose. Fruit sugar (levulose) and milk sugar (lactose) enter the blood unchanged. The blood in the portal vein passing to the liver contains, according to the nature of the food, glycose, lactose, levulose, saccharose, and sometimes traces of dextrin and maltose. All are changed into glycogen in the liver as long as the blood current remains moderate. Muscles and the cells of many glands likewise accumulate glycogen, and by inference from what we know of animals, we may estimate that from 300 to 400 grammes of glycogen are stored away in these structures and the liver in the human organism. If in a human being there is a gradual overfeeding with carbohydrates, the body is taxed with the problem of housing the surplus material elsewhere, and this it accomplishes by converting into fat whatever it is not able to store away in the shape of glycogen; and in this wise every 100 grammes of grape sugar leaves a residue of 39 grammes of fat. But if the surcharge of carbohydrate material is too rapid, the tissues have not time to convert the whole into glycogen or fat and hyperglycæmia, and glycosuria results.

Noorden has found that healthy individuals can invariably take a dose of 180 grammes of grape sugar, of 170 grammes of fruit sugar, of 150 grammes of cane sugar, of 120 grammes of milk sugar on an empty stomach in the morning without producing glycosuria. If we want to obtain in any individual the "limit of assimilation," a larger amount of carbohydrate must be given after an interval of at least two days. If glycosuria ensued after the above-mentioned dose, he regarded the "limit of assimilation" for that individual as sub-normal. Repeated experiments have shown that the limit of assimilation is different for different kinds of sugar; in healthy individuals it is reached on the average when about 200—250 grammes grape sugar, or 200 grammes of fruit sugar, or about 150—200 grammes of cane sugar, or 120 grammes of milk sugar have been taken (variations to 10 or 15 per cent. are not to be regarded as pathological). Noorden has found (1) that normally there is no limit of assimilation for starch, for, however much starch be introduced into the body, no carbohydrate will be found to enter the urine. So much time is taken up in the digestion of the starch that sudden overcrowding of the blood becomes impossible; so that, if a person passes sugar after taking starch he is probably suffering from diabetes mellitus; (2) the sugar found in the urine is invariably of the same kind as that on which the person has been overfed.

It is interesting to note that Noorden found that the limit of assimilation was almost always normal in almost all diseases of the liver, more especially in cirrhosis, in affections of the heart and lungs giving rise to dyspnœa and congestion, in leucæmia, Hodgkin's disease, pernicious anæmia, chlorosis, in fevers, diseases of muscles, including advanced atrophia muscularis, and in diseases of the nervous system, with the exception of Graves' disease, in which the limit of assimilation was, in repeated instances, actually diminished. He refers to his pupil's (Fülger) experiments on the lactosuria of puerperal women, and states that they (Fülger and Noorden) arrived at the following conclusions: (1) The limit of assimilation for lactose is depressed in puerperæ; (2) if grape sugar is administered under the same conditions, milk sugar is frequently passed, but never a trace of grape sugar. This he explains by assuming that in the puerperal condition the faculty for destroying milk sugar is diminished; therefore lactosuria is produced (1) even by those small amounts of milk sugar that are absorbed from the lacteal glands, (2) after the administration of milk sugar more readily than in other women, (3) after the administration of grape sugar, probably because, in the presence of a large amount of grape sugar, even the small amount of lactose normally dealt with in the body is neglected by preference for grape sugar.

[1] Internat. Clinics, Vol. IV., No. 3.

### INFECTIVE DISEASES.

**Typhoid.**—It has been stated that more than 700 papers have been written on the means of identifying the bacillus of typhoid. A simple method has at last been devised by Dr. Elsner,[1] of Koch's Institute. The culture medium is formed of gelatine, infusion of potatoes, and a little potassium iodide spread on plates in the usual way. Every other bacillus besides the typhosus and B. coli dies on this soil. The colonies of the former produce round, clear drops, while the latter grows as large dark brown patches. By this means Elsner is able to detect the disease at all stages, to foretell relapses, and to decide when patients cease to be infective. Lazarus[2] and Brieger have employed this method with success in many cases, and usually obtained definite results in 48 hours. Some precautions in the application of Ehrlich's Diazo test for typhoid have been noted by R. T. Hewlett.[3] First, the sodium nitrite solution must not be more than a day old, and secondly, any excess of the nitrite must be avoided, otherwise a false reaction may occur. Greene even thinks that better results are obtained if only one part of the nitrite is added to a hundred of the sulphanilic acid, instead of the usual one to forty. Again, the colour of the reaction must be a red, not an orange; the froth produced on shaking is pink, not white or colourless, in real cases of typhoid, but the water to be tested must be fresh and acid. Neither test-tube cultivations of the bacillus nor any one of a large number of likely drugs which Hewlett tried fulfilled these requirements, so that he failed to discover anything as to the cause of it. Morphine, indeed, gives a similar reaction, but without the green precipitate on standing which the typhoid poison causes.

On the question of dietary, E. Pritchard[4]

argues that milk as usually given affords too much fat, too little proteid, and only one-quarter of the normal amount of carbohydrates. This, of course, is a large deficiency; but though it is easy to increase the amount of proteid, Pritchard confesses that it is difficult to augment the carbohydrates without irritating the bowels. He would prefer a diet similar to that in measles, but this is impossible in view of intestinal ulcers and other difficulties, unless maltine or other soluble carbohydrates be employed. The Brand system appears to be growing in efficiency. Some time ago they announced statistics showing 7 per cent. mortality, instead of the 14 per cent. met with under expectant treatment. Now, Baruch[5] quotes a number of reports, including more than 5,500 cases in which the mortality was less than 3 per cent., while Brand himself claims that, out of 1,200 recent cases, none died who had received the bath before the fifth day. Among unusual symptoms and complications, W. Russell[6] notices two instances where a rash like that from scarlet fever appeared before the typhoid one, though not followed by sore throat or desquamation. J. M. Day[7] mentions that, when the rash is abundant, the spots may not fade on pressure, and may leave brown stains behind them; while he looks on an ordinary rash, when abundant, as a prognostic of a severe attack. Carter, of Birmingham, reports a case in which, among other curious symptoms, there were hæmoptysis, hæmaturia, ecchymoses, and epistaxis, as well as membranous laryngitis, with ulcers over the arytenoids. No treatment had much effect, and the patient, a strong young man, gradually sank. T. Ch. Simpson[8] records the history of another case in which the disease proved the exciting cause of a hystero-cataleptic condition lasting off and on for many years, together with partial aphasia. The latter was of the type described by Wyllie as specially common after fevers, and was of the slurring character found in such cases. Simpson refers to other instances where cerebral weakness, imbecility, &c., have followed an attack of typhoid. The desirability of performing laparotomy for perforation is pleaded by F. H. Wiggin,[9] who finds recorded only 24 cases, with a successful result in six. He advises that the patient should be first helped over his collapse by strychnia and other stimulants, that ether should then be used as the anæsthetic, or even a local anæsthetic might be employed; and every means should be taken to shorten the time which the operation consumes. A remarkable outbreak of typhoid among French dragoons was traced by Henrot,[10] of Rheims, to their galloping over fields which had been manured with human excreta, much of which had become dust, and was spread in the air by the hoofs of their chargers. In Massachusetts[11] an outbreak occurred through the skim milk sent out from a "creamery," which was infected before distribution, as in the case described some time ago by Dr. J. J. Welply. No instance here occurred of infection conveyed through the butter, and the writer of the report observes that no proof of such infection has ever been obtained. The outbreak at St. Peter's Home, Woking,[12] is said to have followed the arrival of a woman who was convalescent from typhoid. This of course shows the need of some such method as Elsner's for testing the completeness of a patient's recovery. The disease spread in the institution, it was thought, through the failure of the ventilating pipes, and by the anti-syphonage, which took place in some of the drains. Whether enteric is thus conveyed by sewer gas or not, septicæmic conditions do arise in patients who are subject to its influence. Lapthorn Smith, of Montreal,[13] gives a series of instances, where puerperal and surgical patients made unsatisfactory progress, which could be traced to slight defects in the drainage. Indeed, he lays it down that it is far safer to operate in a hovel or building without any drainage than in one where there is any possible passage from the sewers.

**Tetanus.**—The following cases have been treated by antitoxic serum: One under the care of Messrs. A. Turner and Cheatle.[14] This was of eight days' incubation, and the effect of the serum in stopping the spasms was marked and quick. The patient, a boy of six years, made a good recovery. Mr. Farrant reports an unsuccessful case. Here incubation lasted only four days; treatment was commenced on the fifteenth day after onset, but the patient was an alcoholic subject, and sank in spite of some relief from spasm. H. E. Tracey[15] treated successfully a child of seven years of age with Tizzoni's anti-toxin. This was given on the seventh day after the onset. The incubation period was unknown, except that it was under a fortnight. Another case, an unsuccessful one, was treated at Rangoon by O. Baker.[15] The patient, a Mussulman, 17, had an incubation period of less than twelve days, and the antitoxin was injected on the second day after onset, but with no effect. Berger described another unsuccessful case, and in so doing speaks of the almost invariable failure of the antitoxin in France,[16] where only benign chronic tetanus seems to be affected by it. Dr. Macartney[17] treated two cases. One, a woman who developed tetanus eight days after operation, died though treated on the eighth day of her illness by injections of serum. The second, a boy of 5½ years, also died. The incubation here was seven days, and the serum was injected on the day of onset. A third patient, an adult male, had an incubation period of four days, was injected some hours after the onset, and died next day.

A fatal case of cephalic tetanus is reported by E. F. Trevelyan[18] with a good account of the literature of the subject. The incubation period here was less than thirteen days, the injections were commenced on the day after onset, and the fatal termination followed on the fifth day. E. H. Howlett[19] gave an account of a patient who made a good recovery after injection, but the incubation period was very long, though injections were not made till the seventh day of the disease. Another case of recovery is given by R. Hartley,[20] where the incubation was about fourteen days and the treatment was begun on the first day of the disease. Symptoms continued for a fortnight. From these cases it is clear that the efficacy of the remedy is still very limited in all acute cases.

[1] Lancet, Jan. 4. [2] Med. Rec., Feb. 1. [3] Brit. M. J., Jan. 18. [4] The Hospital, Feb. 22. [5] Med. Rec., Jan. 18 [6] Lanc., March 21. [7] Dublin Jour., March. [8] Edinb. Med. J., Jan. [9] Med. Press, Jan. 1. [10] Lancet, Feb. 1. [11] Lancet, Feb. 1. [12] Lancet, Jan. 11. [13] Med. Rec., Feb. 22. [14] Lancet, Dec. 7. [15] Lancet, Feb 1. [15] B.M.J., Jan. 25. [16] Med. Week, Dec. 22. [17] Lancet, Jan. 18. [18] B.M.J., Feb. [19] Med. Chron., Dec. [20] Lancet, Dec. 7.

